# Major depressive disorder plays a vital role in the pathway from gastroesophageal reflux disease to chronic obstructive pulmonary disease: a Mendelian randomization study

**DOI:** 10.3389/fgene.2023.1198476

**Published:** 2023-06-19

**Authors:** Menglong Zou, Wei Zhang, Lele Shen, Yin Xu, Ying Zhu

**Affiliations:** The First Hospital of Hunan University of Chinese Medicine, Changsha, Hunan, China

**Keywords:** instrumental variables, Mendelian randomization, gastroesophageal reflux disease, major depressive disorder, chronic obstructive pulmonary disease, causal effect, expression quantitative trait loci

## Abstract

**Background:** Observational studies have shown a bidirectional association between chronic obstructive pulmonary disease (COPD) and gastroesophageal reflux disease (GERD), but it is not clear whether this association is causal. In our previous study, we found that depression was a hot topic of research in the association between COPD and GERD. Is major depressive disorder (MDD) a mediator of the association between COPD and GERD? Here, we evaluated the causal association between COPD, MDD, and GERD using Mendelian randomization (MR) study.

**Methods:** Based on the FinnGen, United Kingdom Biobank, and Psychiatric Genomics Consortium (PGC) databases, we obtained genome-wide association study (GWAS) summary statistics for the three phenotypes from 315,123 European participants (22,867 GERD cases and 292,256 controls), 462,933 European participants (1,605 COPD cases and 461,328 controls), and 173,005 European participants (59,851 MDD cases and 113,154 controls), respectively. To obtain more instrumental variables to reduce bias, we extracted relevant single-nucleotide polymorphisms (SNPs) for the three phenotypes from published meta-analysis studies. Bidirectional MR and expression quantitative trait loci (eQTL)-MR were performed using the inverse variance weighting method to assess the causal association between GERD, MDD, and COPD.

**Results:** There was no evidence of a causal effect between GERD and COPD in the bidirectional MR analysis [forward MR for GERD on COPD: odds ratios (OR) = 1.001, *p* = 0.270; reverse MR for COPD on GERD: OR = 1.021, *p* = 0.303]. The causal effect between GERD and MDD appeared to be bidirectional (forward MR for GERD on MDD: OR = 1.309, *p* = 0.006; reverse MR for MDD on GERD: OR = 1.530, *p* < 0.001), while the causal effect between MDD and COPD was unidirectional (forward MR for MDD on COPD: OR = 1.004, *p* < 0.001; reverse MR for COPD on MDD: OR = 1.002, *p* = 0.925). MDD mediated the effect of GERD on COPD in a unidirectional manner (OR = 1.001). The results of the eQTL-MR were consistent with those of the bidirectional MR.

**Conclusion:** MDD appears to play a vital role in the effect of GERD on COPD. However, we have no evidence of a direct causal association between GERD and COPD. There is a bidirectional causal association between MDD and GERD, which may accelerate the progression from GERD to COPD.

## 1 Introduction

Chronic obstructive pulmonary disease (COPD) is a chronic and progressive disease that causes a pathological degeneration of the respiratory system (Christenson et al., 2022). It has high morbidity and mortality worldwide and is characterized by airflow limitation and a number of associated comorbidities (Christenson et al., 2022). Between 51% and 88% of those with COPD were reported to have at least one comorbidity, such as arthritis, cardiovascular disease, diabetes, osteoporosis, and chronic pain (Chen et al., 2017; Westerik et al., 2017). The mechanisms responsible for these comorbidities and the exact causal relationship with the presence of COPD are not fully understood (Smith and Wrobel, 2014). The prevalence of osteoporosis in people with COPD is two to five times higher than in people without COPD, which may be related to poor lung function resulting in less improvement in functional exercise capacity (Hornikx et al., 2013; Masala et al., 2014; Sheng et al., 2015). Dyspnea caused by COPD activates common areas of the brain, such as the anterior insula and medial insula, which may alter areas of pain perception, leading to abnormal pain (HajGhanbari et al., 2012). Although some studies analyzed the causes of these comorbidities in COPD, high-quality evidence of genetic cause-and-effect relationships is still needed.

Gastroesophageal reflux disease (GERD) is a condition in which the contents of the stomach back up into the esophagus and throat, causing uncomfortable symptoms and comorbidities that affect several systems, including the digestive, cardiovascular and respiratory systems (Maret-Ouda et al., 2020). The prevalence of GERD ranges from 17% to 54%, of which 58% of cases have no obvious clinical symptoms of reflux other than chronic cough (Shimizu et al., 2012; Smith and Wrobel, 2014). For this reason, the role of GERD in the pathogenesis of chronic lung disease has received much attention in the last decade. Reflux of gastric contents is known to cause significant irritation and damage to the airways, increasing changes in bronchial reactivity and leading to pulmonary symptoms (Ours et al., 1999; Sontag, 2005). In addition, medications such as β-agonists lower the tone of the lower esophageal sphincter and diminish the function of the anti-reflux barrier in patients with COPD, which provides an opportunity for reflux of gastric contents (Shimizu et al., 2012; Del Grande et al., 2016).

Major depressive disorder (MDD) is a mental illness characterized by loss of interest, depression and cognitive impairment (Otte et al., 2016). In recent years, MDD has become a serious public mental health problem, affecting around 264 million people worldwide (Otte et al., 2016; GBD, 2017 Disease and Injury Incidence and Prevalence Collaborators, 2018). Mental disorders can worsen the symptoms of GERD by increasing the secretion of stomach acid, and GERD can also increase the risk of mental disorders (Naliboff et al., 2004; Kim et al., 2018; Chen et al., 2023). Furthermore, depression is a risk factor for worsening COPD and increased mortality in COPD patients (Salte et al., 2015; Iyer et al., 2016). Our previous study found that depression is a research hotspot for the association between COPD and GERD (Zou et al., 2022). On the basis of these reports, we propose the hypothesis that MDD may play an important role in the association between COPD and GERD.

Mendelian randomization (MR) is a novel method of epidemiological research that uses genetic variants as instrumental variables to assess the presence or absence of causal effects between exposure and outcome (Burgess et al., 2019). Evidence of causal effects analyzed in this method greatly reduces the bias caused by confounders in observational studies, because the genetic variants are randomly assigned at the time of conception and are unaffected by environmental factors and self-selected lifestyle (Smith and Ebrahim, 2004). An MR study performed by Han et al. to assess the effect of white blood cell count on COPD showed that a high blood eosinophil count increased the risk of COPD (Han et al., 2022). Another MR research by Higbee et al. found no evidence of a causal relationship between COPD and cognitive decline (Higbee et al., 2021). Reassuringly, large-scale genome-wide association study (GWAS) summary statistic data on GERD, MDD, and COPD have been published publicly, providing an opportunity to sort out the complex causal relationships between them through MR analysis. Here, we would like to explore whether MDD plays a mediator role in the causal relationship between COPD and GERD through bidirectional MR analysis. In addition, single-nucleotide polymorphisms (SNPs) related with mRNA expression, also known to as expression quantitative trait loci (eQTL), were used as new instrumental variables for eQTL-MR to validate the robustness of the results.

## 2 Materials and methods

### 2.1 Study design

**FIGURE 1:**
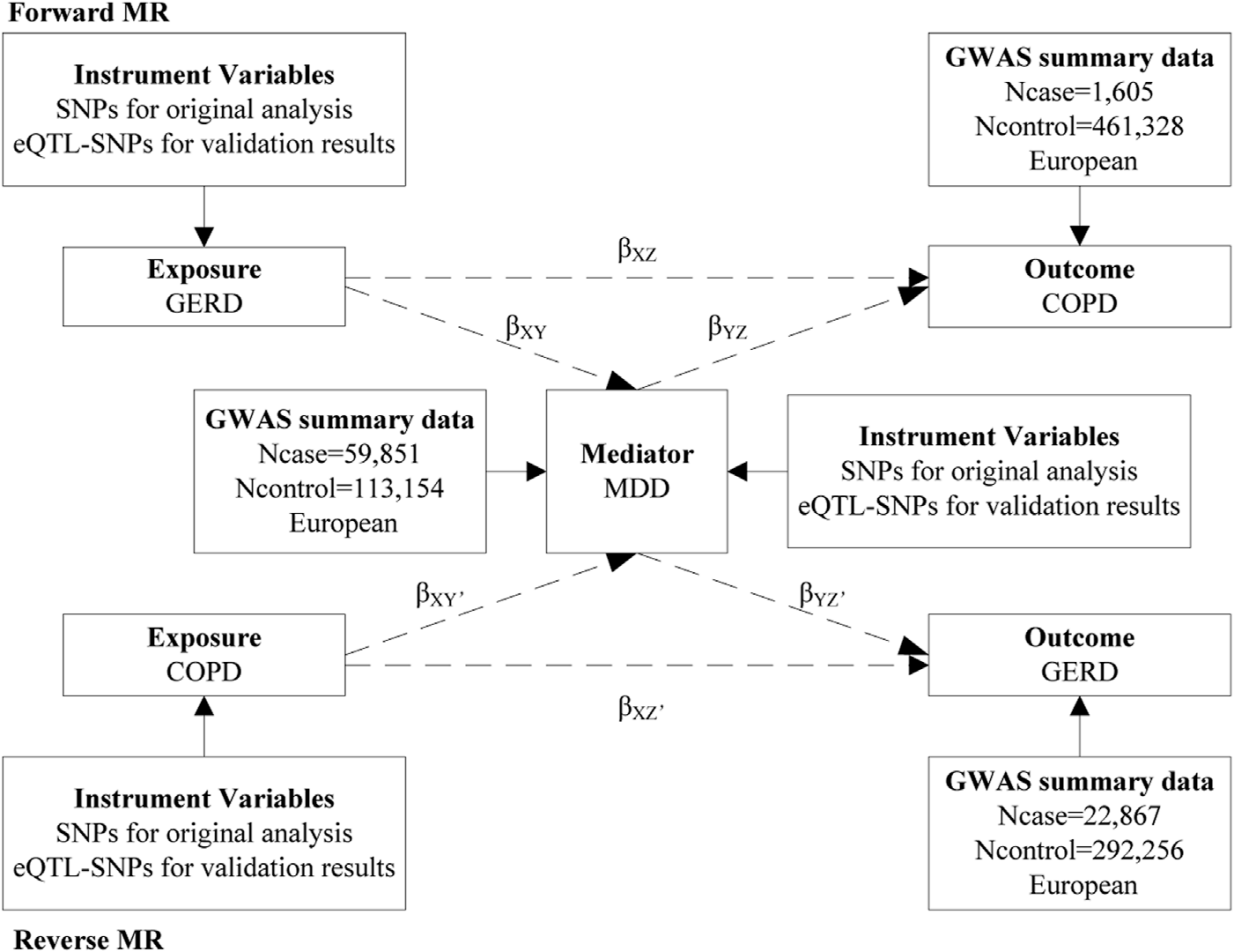
The present study design overview. MR, mendelian randomization; SNPs, single-nucleotide polymorphisms; eQTL, expression quantitative trait loci; GERD, gastroesophageal reflux disease; GWAS, genome-wide association study; COPD, chronic obstructive pulmonary disease; MDD, major depressive disorder.

A bidirectional, two-step MR study was performed to assess the role of MDD in the association between GERD and COPD (Figure 1). In the forward MR analysis (if there is a causal association), the direct effect of GERD on COPD is equal to *β*_*XZ*_, the indirect effect of GERD on COPD via MDD is equal to *β*_*XY*_ *β*_*YZ*_, and the total effect is equal to *β*_*XZ*_ + *β*_*XY*_ *β*_*YZ*_ (Sanderson, 2021; Zhou et al., 2021). Confidence intervals for the indirect effect were estimated using the delta method. In the reverse MR analysis, we used the same formula for calculations. The stability of the results was assessed by using the eQTL-based MR. The present study follows the STROBE-MR guidelines (Skrivankova et al., 2021). Ethical approval was not required as we used publicly available GWAS results from relevant publications and databases in this study.

### 2.2 Instrumental variables selection

The genetic instrumental variables for three phenotypes should meet the three main assumptions (Davies et al., 2018). First, instrumental variables should be strongly associated with corresponding phenotype. Second, instrumental variables should be unaffected by potential confounders of the exposure-outcome association. Third, direct links between the instrumental variables and the outcome are not available. To minimize bias, we extracted SNPs for the three phenotypes directly from large GWAS meta-analysis literature (the largest number of cases of each phenotype to date). The SNPs selection for GERD, COPD, and MDD was based on meta-analysis of five GWAS with 385,276 European-descent participants (80,265 GERD cases and 305,011 controls) (An et al., 2019), genome-wide association meta-analysis of up to 257,811 European-descent participants (35,735 COPD cases and 222,076 controls) (Sakornsakolpat et al., 2019), and meta-analysis of three large GWAS with 807,553 European-descent participants (246,363 MDD cases and 561,190 controls) (Howard et al., 2019), respectively. The genome-wide statistical significance threshold (*p* < 5 × 10^−8^) was met for all SNPs associated with GERD, COPD and MDD to fulfil assumption 1. The parameters (*r* ^2^ < 0.001 and clump window = 10,000 kb) were set for linkage disequilibrium (LD) clumping to ensure those SNPs were independent. We excluded SNPs that were strongly associated with confounders for the purposes of following assumption 2. With the SNPs meeting assumptions 1 and 2, we then proceeded to check for assumption 3. Data on exposure and outcome were merged and then harmonized by effect allele. We examined the *p*-value of each SNP in the outcome data. The SNPs with *p* < 5 × 10^−5^ in the outcome data were discarded because of a possible violation of assumption 3. In addition, we also removed incompatible SNPs or palindromic SNPs. The eQTL-SNPs were collected by analyzing SNPs in the Phenoscanner database (http://www.phenoscanner.medschl.cam.ac.uk/) with *p* < 5 × 10^−8^. We calculated the R statistic [*R* ^2^ = 2 × EAF × (1-EAF) × beta^2^, where EAF is the effect allele frequency and beta is the estimate of the effect of the SNP] for each SNP to detect the proportion of variation (Palmer et al., 2012). Subsequently, the F statistic [F = *R* ^2^ × (N−2)/(1−R^2^), where N is the sample size] was used to assess the bias of the weak instrumental variables (Palmer et al., 2012). When the F-statistic >10, the bias of weak instrumental variables can be ignored (Burgess et al., 2011).

### 2.3 Data source

In MR analysis, sample overlap from different large GWAS meta-analysis is a potential source of bias. In addition, summary statistics are not readily available for some large GWAS meta-analysis (Au Yeung et al., 2022). Considering these issues, the summary statistics used in this study were not derived from the three large meta-analysis studies mentioned above. The different data sources are comparable in terms of genetic association, as they are all of European ancestry (Au Yeung et al., 2022). Summary statistic data for MDD was available from Psychiatric Genomics Consortium (PGC) database, which included a total of 173,005 European-descent participants (59,851 MDD cases and 113,154 controls) (Wray et al., 2018). It is important to note that 29,740 of the 173,005 participants were from the United Kingdom Biobank database, whose cohort information can be found in the original literature. Summary statistic data for COPD was available from the United Kingdom Biobank, including 462,933 European-descent participants (1,605 cases and 461,328 controls) (Bycroft et al., 2018). Despite the potential for sample overlap in the summary statistic data for COPD and MDD, the resulting bias is deemed negligible as the sample overlap is a maximum of 6.4% (Burgess et al., 2016). Summary statistics for GERD were available from the FinnGen database, which included 315,123 European participants (22,867 cases and 292,256 controls) (Mitja et al., 2022).

### 2.4 Confounders analysis

Based on the results of previous bibliometric study, smoking, body mass index (BMI), and standing height are potential confounders of the GERD-COPD association (Zou et al., 2022). We evaluated the association between GERD-SNPs and potential confounders using relevant GWAS, i.e., smoking initiation (*n* = 607,291 from GSCAN), BMI (*n* = 681,275 from GIANT), and standing height (*n* = 461,950 from MRC-IEU), based on the standard error delta method and random effects meta-analysis (Au Yeung et al., 2022). Subsequently, we removed instrumental variables from the GERD-SNPs that were strongly associated with confounders to correct for the pleiotropy of the results.

### 2.5 Genetic correlation analysis

Linkage disequilibrium score regression (LDSC) is a powerful method for genetic correlation analysis of complex diseases or traits by calculating the heritability of a trait based on SNPs or the correlation of two traits based on SNPs using Chi-squared statistics (Tobin et al., 2004; Xu et al., 2022). In this study, we investigated the genetic correlation between GERD, MDD, and COPD using LDSC method. The Bonferroni-corrected *p*-value of 0.017 (0.05/3) was set as the threshold of statistical significance, considering that we performed three genetic correlation analysis. Suggestive genetic association results were those that were significant (*p* < 0.05) before but not after multiple-comparison correction (*p* < 0.017).

### 2.6 MR analysis

According to the MR analysis guidelines (Burgess et al., 2013), the inverse variance weighting (IVW) method is statistically most effective. Therefore, the IVW method was used as the primary analytical method to further investigate the genetic correlation results identified by LDSC. Three additional MR methods (MR-Egger regression, Weighted median, and Maximum likelihood) were used as a complement. MR results were expressed as odds ratios (OR) with 95% confidence intervals (CI).

In addition, we performed multiple sensitivity analysis to check the robustness of the MR results, such as Cochran’s Q test, MR-Egger intercept test, MR-PRESSO, leave-one-out analysis, and MR-Steiger test of directionality. Cochran’s Q is a test for heterogeneity and a significant *p*-value indicates the presence of heterogeneity (Higgins et al., 2003; Bowden et al., 2017). MR-Egger intercept analysis was performed to assess horizontal pleiotropy (Bowden et al., 2015). The MR analysis was re-conducted after removing the outliers (if applicable) identified by the MR-PRESSO (Verbanck et al., 2018). Leave-one-out analysis was conducted to identify if any single SNPs had a disproportionate effect on the estimates (Verbanck et al., 2018). The MR-Steiger directionality test was performed to evaluate the validity of the direction of causality (Hemani et al., 2017). Considering that our study included three diseases and that the MR analysis was bidirectional, a Bonferroni-corrected *p*-value of 0.008 (0.05/6) was set as the threshold for statistical significance. Suggestive association results for MR analysis were those that were significant (*p* < 0.05) before but not after multiple-comparison correction (*p* < 0.008).

### 2.7 Functional mapping and annotation (FUMA) analysis

FUMA is an online analysis platform that can be used to annotate and visualize GWAS results (Watanabe et al., 2017). To further investigate the genetic mechanisms underlying the association between GERD, MDD, and COPD, we performed a FUMA analysis. We extracted gene symbols corresponding to eQTL from the Phenoscanner database. Subsequently, we annotated these gene symbols through the GENE2FUNC function of the FUMA platform.

MR analysis and sensitivity analysis in our study were performed using the package “TwoSampleMR (version 0.5.6)” and the package “MRPERSSO (version 1.0)” in the Rstudio (R version 4.2.2). Genetic correlation analysis was performed using the package “ldscr (version 0.1.0).” FUMA analysis was performed using an online tool (https://fuma.ctglab.nl/).

## 3 Results

### 3.1 Selection of instrumental variables

A two-step, bidirectional MR analysis was performed to explore the role of MDD in the GERD-COPD association. As presented in Supplementary Tables S1–S6, the mean F-statistic was 223 (ranging from 146 to 321) for GERD, 148 (ranging from 23 to 432) for MDD, and 713 (ranging from 79 to 4,318) for COPD. The F-statistics for all instrumental variables were greater than 10, indicating a low risk of bias due to weak instrumental variables in this study. In the forward MR analysis, 18 SNPs (14 SNPs were considered to be eQTL-SNPs) for GERD on COPD (Supplementary Table S1), 17 SNPs (14 SNPs were considered to be eQTL-SNPs) for GERD on MDD (Supplementary Table S2), and 56 SNPs (37 SNPs were considered to be eQTL-SNPs) for MDD on COPD (Supplementary Table S3) were selected as instrumental variables. In the reverse MR analysis, 54 SNPs (37 SNPs were considered to be eQTL-SNPs) for COPD on GERD (Supplementary Table S4), 53 SNPs (36 SNPs were considered to be eQTL-SNPs) for COPD on MDD (Supplementary Table S5), and 68 SNPs (46 SNPs were considered to be eQTL-SNPs) for MDD on GERD (Supplementary Table S6) were selected as instrumental variables. GERD-increasing alleles were positively associated with smoking and BMI, and inversely associated with standing height (Supplementary Table S7). According to genome-wide significance (*p* < 5 × 10^−8^), we identified 93, 507, and 773 strongly associated SNPs from smoking initiation, BMI and standing height GWAS data, respectively (Supplementary Table S8).

### 3.2 Genetic correlation analysis

LDSC analysis identified three genetic correlations between the three traits of GERD, MDD, and COPD, such as GERD and MDD (genetic correlation = 0.449, *p* = 3.88 × 10^−21^), GERD and COPD (genetic correlation = 0.479, *p* = 4.44 × 10^−5^), and MDD and COPD (genetic correlation = 0.562, *p* = 3.68 × 10^−9^).

### 3.3 Forward MR finding

**FIGURE 2:**
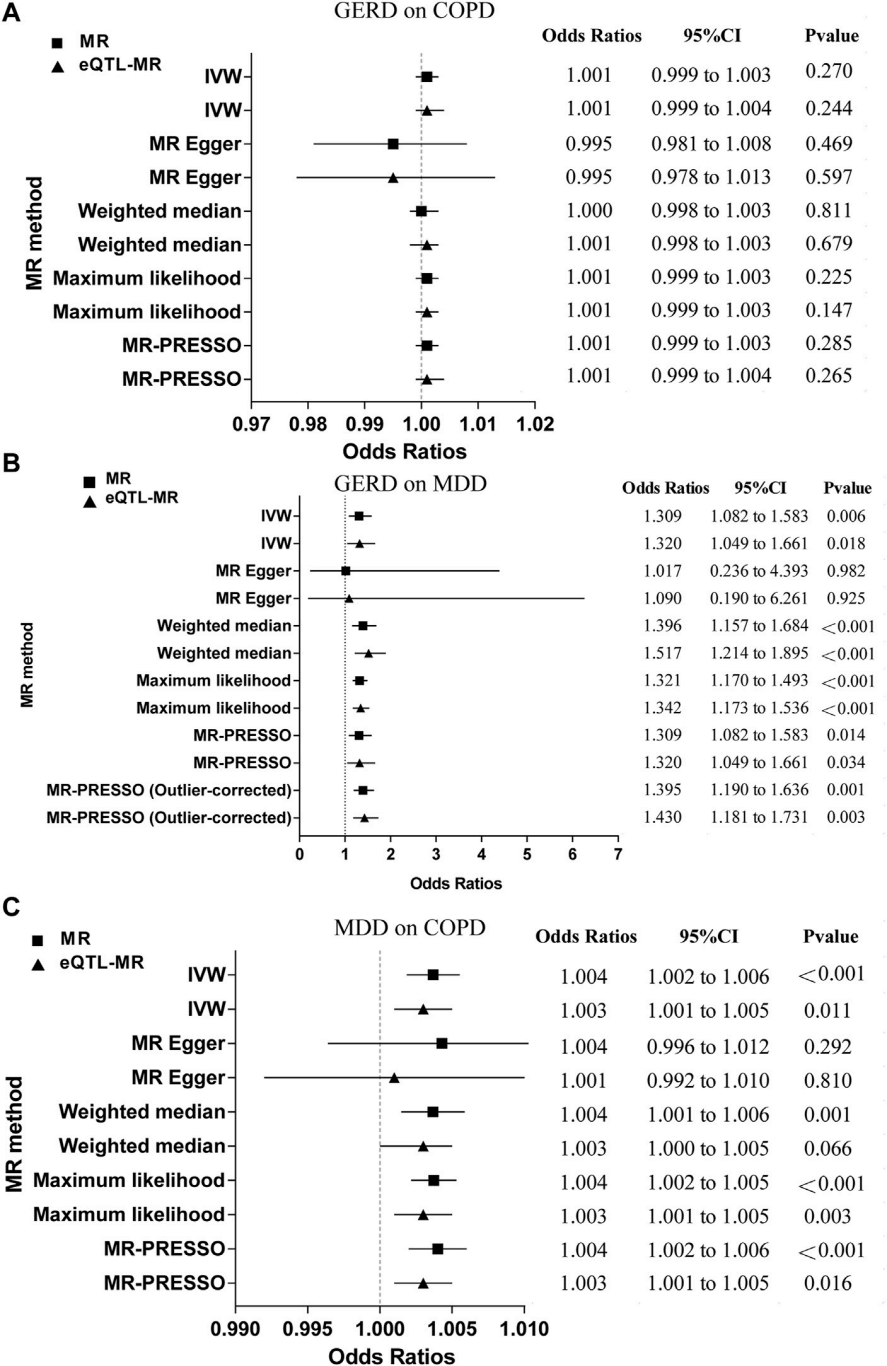
The MR results for the forward analysis. **(A)** GERD on COPD; **(B)** GERD on MDD; **(C)** MDD on COPD. MR, mendelian randomization; eQTL, expression quantitative trait loci; GERD, gastroesophageal reflux disease; COPD, chronic obstructive pulmonary disease; MDD, major depressive disorder; IVW, inverse variance weighting; MR-PRESSO, mendelian randomization pleiotropy residual sum and outlier; CI, confidence intervals.

Figure 2A; Supplementary Table S9 showed that there was no evidence of a causal association of genetically predicted GERD on COPD (OR = 1.001; 95% CI = 0.999 to 1.003, *p* = 0.270). The results were consistent with the estimations made in the MR-Egger, Weighted median, Maximum likelihood, or MR-PRESSO. The MR-PRESSO method did not detect any outliers that affected the results. The scatter plot visualized the effect of individual SNP (Supplementary Figure S1A), and the forest plot showed the causal association of each GERD-SNP on COPD (Supplementary Figure S2A). The MR-Egger intercept test did not reveal the presence of horizontal pleiotropy, and the Cochran’s Q test did not indicate the presence of heterogeneity (Supplementary Table S13). The MR-Steiger directionality test showed that the estimated variance explained was much greater for GERD than for COPD, indicating that the observed direction of causality was likely to be correct (Supplementary Table S15). Furthermore, the leave-one-out analysis did not find that the MR estimates were driven by a single specific SNP (Supplementary Figure S3A), and the funnel plot generated by the IVW method showed a symmetric distribution of the effect of GERD on COPD (Supplementary Figure S4A), indicating the robustness of the results. The eQTL-SNPs associated with GERD were used as new exposure data to re-perform MR analysis to validate the robustness of the results. We observed similar results (OR = 1.001; 95% CI = 0.999 to 1.004, *p* = 0.244) (Figure 2A; Supplementary Table 10). The results were consistent with the estimations made in the MR-Egger, Weighted median, Maximum likelihood, or MR-PRESSO. In the forward eQTL-MR analysis for GERD on COPD, the MR-PRESSO method also detected no outliers that affected the results. As expected, the sensitivity analysis was also consistent with the original MR analysis for GERD on COPD, which included the scatter plot for the effect of individual eQTL-SNP (Supplementary Figure S1D), the forest plot for the causal association of each GERD-related eQTL-SNP on COPD (Supplementary Figure S2D), the MR-Egger intercept test (Supplementary Table S14), the Cochran’s Q test (Supplementary Table S14), the MR-Steiger directionality test (Supplementary Table S16), the leave-one-out analysis (Supplementary Figure S3D), and the funnel plot for distribution of the effect of GERD on COPD (Supplementary Figure S4D), indicating strong robustness of the results.

Figure 2B; Supplementary Table S9 revealed that genetically driven GERD on MDD was a significant causal estimate (OR = 1.309; 95% CI = 1.082 to 1.583, *p* = 0.006). Results were consistent with estimates made using the Weighted median, Maximum likelihood, or MR-PRESSO. The individual SNP effects were visualized in the scatter plot (Supplementary Figure S1B), and the forest plot showed the causal association of each GERD-related SNP on MDD (Supplementary Figure S2B). Although the Cochran’s Q-test revealed the presence of heterogeneity (Supplementary Table S13), the MR-Egger intercept test did not indicate the presence of horizontal pleiotropy (Supplementary Table S13) and the leave-one-out analysis did not suggest that MR estimates were driven by a single specific SNP (Supplementary Figure S3B). Furthermore, the causal effect of the results remained significant after discarding the outliers identified by MR-PRESSO (OR = 1.395; 95% CI = 1.190 to 1.636, *p* = 0.001). Based on the results of the MR-Steiger directionality test, it was found that the variance explained in GERD was much greater than that in MDD, indicating the observed causal direction was likely to be accurate (Supplementary Table S15). The funnel plot obtained by the IVW method showed a symmetric distribution of the effect of GERD on MDD, indicating the robustness of the results (Supplementary Figure S4B). We observed similar results in the eQTL-MR analysis (OR = 1.320; 95% CI = 1.409 to 1.661, *p* = 0.018) (Figure 2B; Supplementary Table S10). Results were consistent with those obtained using Weighted median, Maximum likelihood, or MR-PRESSO estimations (Figure 2B; Supplementary Table S10). After discarding MR-PRESSO-identified outliers in forward eQTL-MR analysis for GERD on MDD, the causal effect remained significant (OR = 1.430; 95% CI = 1.181 to 1.731, *p* = 0.003). Similarly, the sensitivity analysis for GERD on MDD in the eQTL-MR analysis was consistent with the original MR analysis, which included the scatter plot for the effect of individual eQTL-SNP (Supplementary Figure S1E), the forest plot for the causal association of each GERD-related eQTL-SNP on MDD (Supplementary Figure S2E), the MR-Egger intercept test (Supplementary Table S14), the Cochran’s Q test (Supplementary Table S14), the MR-Steiger directionality test (Supplementary Table S16), the leave-one-out analysis (Supplementary Figure S3E), and the funnel plot for distribution of the effect of GERD on COPD (Supplementary Figure S4E), indicating strong robustness of the results.

Figure 2C; Supplementary Table S9 indicated that genetic liability to MDD on COPD was a significant causal estimate (OR = 1.004; 95% CI = 1.002 to 1.006, *p* < 0.001). According to the findings, the estimates made with Weighted medians, Maximum likelihoods, or MR-PRESSOs were similar. We found no outliers that affected the results using the MR-PRESSO method. In the scatter plot, the effect of individual SNP was visualized (Supplementary Figure S1C), and in the forest plot, each MDD-related SNP was visualized according to its causal association with COPD (Supplementary Figure S2C). In spite of the presence of heterogeneity indicated by the Cochran’s Q test (Supplementary Table S13), the MR-Egger intercept did not reveal the presence of horizontal pleiotropy (Supplementary Table S13) and the leave-one-out analysis did not find that MR estimates were driven by a single specific SNP (Supplementary Figure S3C). As demonstrated by the MR-Steiger directionality test, MDD had higher variance explained compared to COPD, indicating that causality was likely to follow the observed direction (Supplementary Table S15), and the funnel plot generated by the IVW method showed a symmetric distribution of the effect of MDD on COPD, indicating the robustness of the results (Supplementary Figure S4C). We observed the same trend in the eQTL-MR analysis (OR = 1.003; 95% CI = 1.001 to 1.005, *p* = 0.011) (Figure 2C; Supplementary Table S10). In accordance with Maximum likelihood or MR-PRESSO estimations, the results were similar. The MR-PRESSO method did not detect any outliers in forward eQTL-MR analysis for MDD on COPD. The Cochran’s Q test did not indicate the presence of heterogeneity (Supplementary Table S14). The other sensitivity analysis in the eQTL-MR analysis also supported the MR analysis, which included the scatter plot for the effect of individual eQTL-SNP (Supplementary Figure S1F), the forest plot for the causal association of each MDD-related eQTL-SNP on COPD (Supplementary Figure S2F), the MR-Egger intercept test (Supplementary Table S14), the MR-Steiger directionality test (Supplementary Table S16), the leave-one-out analysis (Supplementary Figure S3F), and the funnel plot for distribution of the effect of MDD on COPD (Supplementary Figure S4F), indicating strong robustness of the results.

Overall, there is no direct causal association of genetic liability to GERD on COPD, but there is an indirect causal association of genetic liability to GERD on COPD via MDD (OR = 1.001; 95% CI = 0.951–1.054).

### 3.4 Reverse MR finding

**FIGURE 3:**
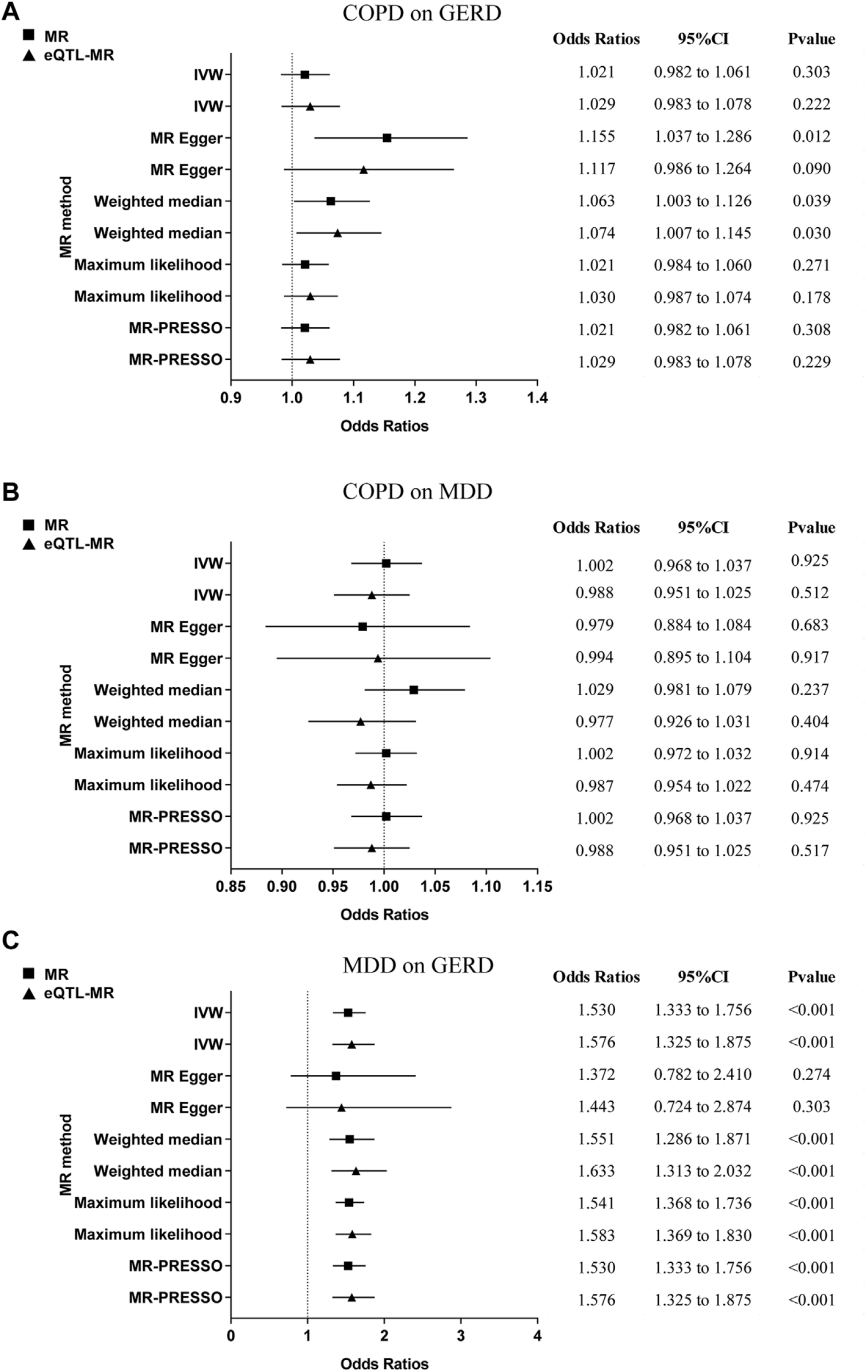
The MR results for the reverse analysis. **(A)** COPD on GERD; **(B)** COPD on MDD; **(C)** MDD on GERD. MR, mendelian randomization; eQTL, expression quantitative trait loci; GERD, gastroesophageal reflux disease; COPD, chronic obstructive pulmonary disease; MDD, major depressive disorder; IVW, inverse variance weighting; MR-PRESSO, mendelian randomization pleiotropy residual sum and outlier; CI, confidence intervals.

There was no significant causal effect for either COPD-GERD (MR: OR = 1.021; 95% CI = 0.982 to 1.061, *p* = 0.303; eQTL-MR: OR = 1.029; 95% CI = 0.983 to 1.078, *p* = 0.222) or COPD-MDD (MR: OR = 1.002; 95% CI = 0.968 to 1.067, *p* = 0.925; eQTL-MR: OR = 0.988; 95% CI = 0.951 to 1.025, *p* = 0.512) in both the reverse MR analysis and the eQTL-MR analysis (Figures 3A, B; Supplementary Tables S11, S12). Figure 3C revealed that genetically predicted MDD on GERD was a significant causal estimate (MR: OR = 1.530; 95% CI = 1.333 to 1.756, *p* < 0.001; eQTL-MR: OR = 1.576; 95% CI = 1.325 to 1.875, *p* < 0.001). The scatter plots of the causal association are shown in Supplementary Figures S5A–F; the forest plots of individual SNP causal effects are shown in Supplementary Figures S6A–F; the leave-one-out plots are shown in Supplementary Figures S7A–F, and the funnel plots are shown in Supplementary Figures S8A–F. In the reverse MR analysis, although potential pleiotropy and heterogeneity were observed (Supplementary Tables S13, S14), no outliers were identified by MR-PRESSO and the MR-Steiger directionality test suggested that the observed directions were true (Supplementary Tables S11, S12, S15, S16).

In conclusion, there is no direct causal association of genetic susceptibility to COPD on GERD, and there is no evidence for an indirect causal association of genetically driven COPD on GERD via MDD.

### 3.5 FUMA analysis

**FIGURE 4:**
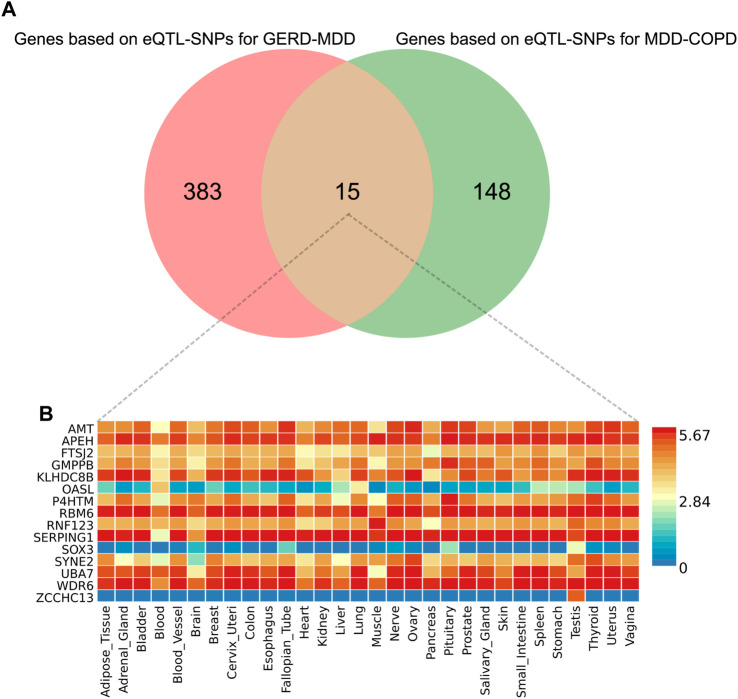
The results of FUMA analysis. **(A)** Venn diagram of overlapping genes; **(B)** Expression of overlapping genes in 30 general tissues. eQTL, expression quantitative trait loci; GERD, gastroesophageal reflux disease; COPD, chronic obstructive pulmonary disease; MDD, major depressive disorder.

We obtained a total of 398 genes based on eQTL-SNPs for GERD-MDD and 163 genes based on eQTL-SNPs for MDD-COPD from the Phenoscanner database. Fifteen genes were repeatedly annotated in these two processes (Figure 4A). Expression of the fifteen genes in 30 general tissues can be found in Figure 4B; Supplementary Table S17. Gene set enrichment analysis (GSEA) was performed to explore possible biological mechanisms of 15 genes associated with GERD-MDD-COPD. A total of 35 gene sets with an adjusted *p* < 0.05 were identified (Supplementary Table S18). We found strong enrichment signals associated with extremely high intelligence (adjusted *p* = 5.55 × 10^−10^). In addition, we also found enrichment of signals related to lifestyle habits such as regular attendance at a religious group (adjusted *p* = 5.55 × 10^−10^ and regular attendance at a gym or sports club (adjusted *p* = 2.06 × 10^−9^).

## 4 Discussion

**FIGURE 5:**
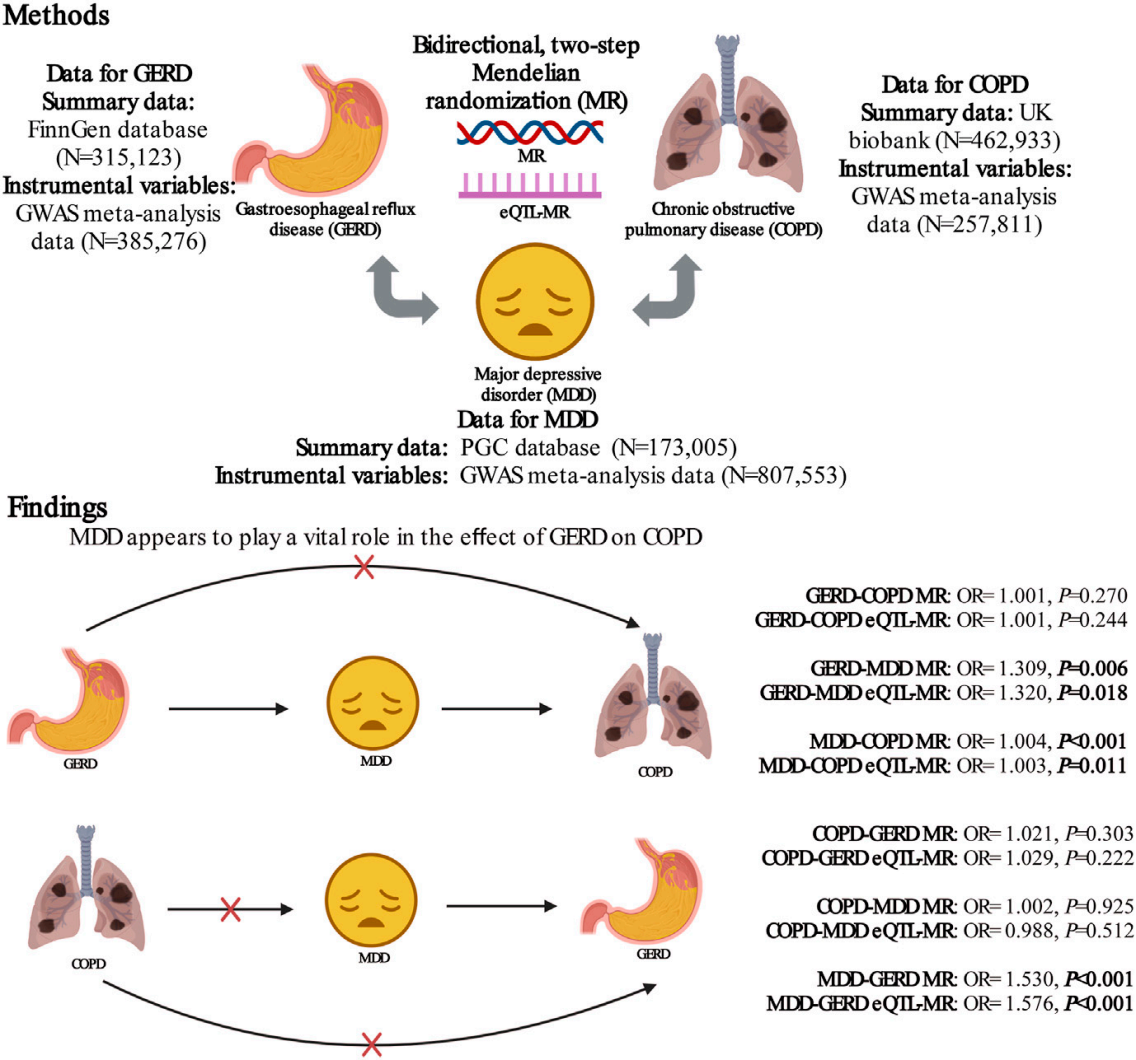
The role of major depressive disorder in the relationship between gastroesophageal reflux disease and chronic obstructive pulmonary disease. MR, mendelian randomization; GERD, gastroesophageal reflux disease; COPD, chronic obstructive pulmonary disease; MDD, major depressive disorder; PGC, Psychiatric Genomics Consortium; GWAS, genome-wide association study. Created with BioRender.com.

In this study, a two-step, bidirectional MR method was used to demonstrate that MDD plays the mediator role in the effect of GERD on COPD (Figure 5). Importantly, these results were also validated by several sensitivity analysis and eQTL-based MR. Although LDSC analysis suggested a genetic correlation between GERD and COPD, our study did not provide strong evidence of a direct causal relationship between GERD and COPD. In addition, we identified 15 genes via FUMA analysis, and these findings contributed to a better understanding of the progression of GERD-MDD-COPD.

A large cross-sectional survey previously conducted by Choi et al. also examined the relationship between GERD and depression, recruiting 19,099 participants (Choi et al., 2018). The diagnosis of GERD in their study was based on the results of upper gastrointestinal endoscopy and the severity of depression was assessed according using the beck depression inventory. After correcting for confounders, the study found that GERD increased the risk of depression, similar to what we found. Another case-control study from the health improvement network UK primary care database of 3,064 GERD patients and 10,000 controls also showed that a diagnosis of depression was associated with an increased risk of a subsequent GERD diagnosis (Martín-Merino et al., 2010). However, in contrast to cross-sectional findings, contradictory results were obtained based on prospective study. The study by Neto et al. classified 245 patients as GERD+ (*n* = 136) and GERD- (*n* = 109) and found similar depression scores in both groups (Neto et al., 2019). The discrepancy between prospective and cross-sectional studies may be attributed to bias caused by confounders. It should be noted that there are also some limitations to MR analysis and that the results may be affected by collider bias, pleiotropy, and outliers. A report by Zeng et al. also used the MR approach to examine the relationship between GERD and depression (Zeng et al., 2023). They concluded that depression did not affect GERD, while GERD increased the risk of depression. Although the authors considered the effects of confounders and pleiotropy, they did not assess the bias in the results caused by outliers. Of course, we cannot rule out the possibility that our study included severe depression and theirs did not mention the level of depression. Overall, we excluded potential confounders more reasonably in our MR analysis based on the results of previous studies and analyzed the bias of outliers to the MR results. More importantly, our results were also validated using eQTL-SNPs, which provided stronger evidence for these results. In our study, we were primarily concerned with the possible mediating role of MDD. Although previous studies have shown that COPD increases the risk of depression (Catalfo et al., 2016; Pollok et al., 2019), which is inconsistent with our findings, we could not exclude that we were only considering the genetic instruments of COPD and not the influence of the diagnosis of COPD. Of course, as mentioned above, we were looking at MDD rather than mild depression, and this may have been one of the reasons. We also identified 15 overlapping genes (such as SERPING1) and 35 gene sets by FUMA analysis. SERPING1 was found to be a specific immunomodulatory mediator in acute exacerbations of COPD (Shi et al., 2018). When interpreting these enrichment results, it is important to note that the FUMA analysis attempts to identify the most likely causal genetic variants and genes, without distinguishing whether the causality is expressed through genes or not (Dai et al., 2022). In conclusion, we could not find strong evidence for an effect of COPD on the risk of MDD, but we found that MDD acts as a mediator to mediate the causal effect of GERD on COPD.

Based on genetic instruments obtained from large GWASs of GERD, MDD and COPD, this study comprehensively investigated the role of MDD in the causal effects of GERD and COPD using a bidirectional two-step MR analysis design. A series of sensitivity analysis and the eQTL-SNPs MR were used to validate the robustness of the results. Nevertheless, some limitations need to be considered when interpreting our findings. First, although we identified several genes associated with the progression of GERD-MDD-COPD, we were unable to directly compare the expression of the identified genes between patients and controls due to a lack of relevant gene expression data. Future studies will need to investigate gene expression changes in the identified genes and further explore possible pathogenic mechanisms. Second, the Cochran’s Q test suggested that there was heterogeneity in the results, which may be an inevitable bias due to the GWAS data sourced from a meta-analysis. To address this issue, we selected random-effects IVW as the primary method for MR analysis, which provided robust results. In addition, discarding outliers (if detected) did not eliminate the causal relationships identified in the original IVW analysis. The results of the MR-Steiger directionality test provided further support for the causal effects found in the MR analysis. Third although we excluded some confounders that were derived from the results of our previous bibliometric analysis (Zou et al., 2022) we might ignore other unknown confounders. In addition, when assessing the effect of GERD on smoking by random-effects meta-analysis, we only analyzed GWAS data on smoking initiation and did not consider smoking frequency. We also did not perform bidirectional MR to identify whether the pleiotropy due to confounders was vertical or horizontal (Au Yeung et al., 2022). Finally, the GWAS results are based on data from European descent, so they may not be representative of the entire population.

## 5 Conclusion

In summary, our findings suggest that MDD has a significant impact on the GERD-COPD pathway, providing evidence to support the idea that MDD may be a potential target for irreversible limitation of airflow due to long-term gastric content reflux-induced esophageal disease. Future research should be performed to investigate the mechanisms behind these cause-and-effect relationships to reduce the burden of disease caused by COPD in patients with GERD.

## Data Availability

Publicly available datasets were analyzed in this study. The data of major depressive disorder (ID: ieu-a-1188) and chronic obstructive pulmonary disease (ID: ukb-b-13447) can be obtained from https://gwas.mrcieu.ac.uk/. The data of gastroesophageal reflux disease (ID: Finngen-R8-IK11-REFLUX) can be obtained from https://www.finngen.fi/en. Any other data generated in the analysis process can be requested from the corresponding authors.
