# Supplementary material for: Major depressive disorder plays a vital role in the pathway from gastroesophageal reflux disease to chronic obstructive pulmonary disease: a Mendelian randomization study
Source: Front Genet. 2023 Jun 19;14:1198476. doi: 10.3389/fgene.2023.1198476 (PMC10315650; doi:10.3389/fgene.2023.1198476)
Supplement: Supplementary file 2 [file Image1.pdf]

Supplementary Figure 1. Scatter plot for the forward MR analysis.
Supplementary Figure 2. Forest plot for the forward MR analysis.
Supplementary Figure 3. Leave-one-out analysis for the forward MR analysis.
Supplementary Figure 4. Funnel plot for the forward MR analysis.
Supplementary Figure 5. Scatter plot for the reverse MR analysis.
Supplementary Figure 6. Forest plot for the reverse MR analysis.
Supplementary Figure 7. Leave-one-out analysis for the reverse MR analysis.
Supplementary Figure 8. Funnel plot for the reverse MR analysis.

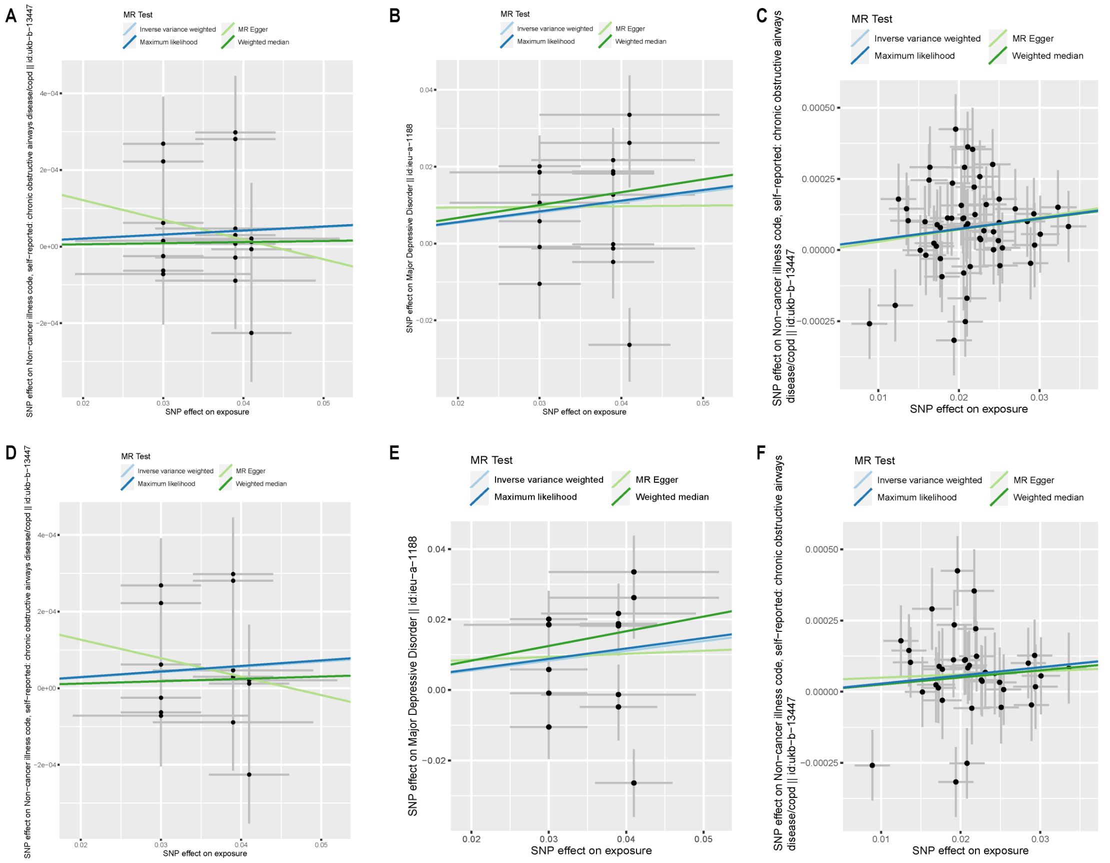

Supplementary Figure 1. Scatter plot for the forward MR analysis. **(A)** GERD-COPD in MR analysis. **(B)** GERD-MDD in MR analysis. **(C)** MDD-COPD in MR analysis. **(D)** GERD-COPD in eQTL-MR analysis. **(E)** GERD-MDD in eQTL-MR analysis. **(F)** MDD-COPD in eQTL-MR analysis.

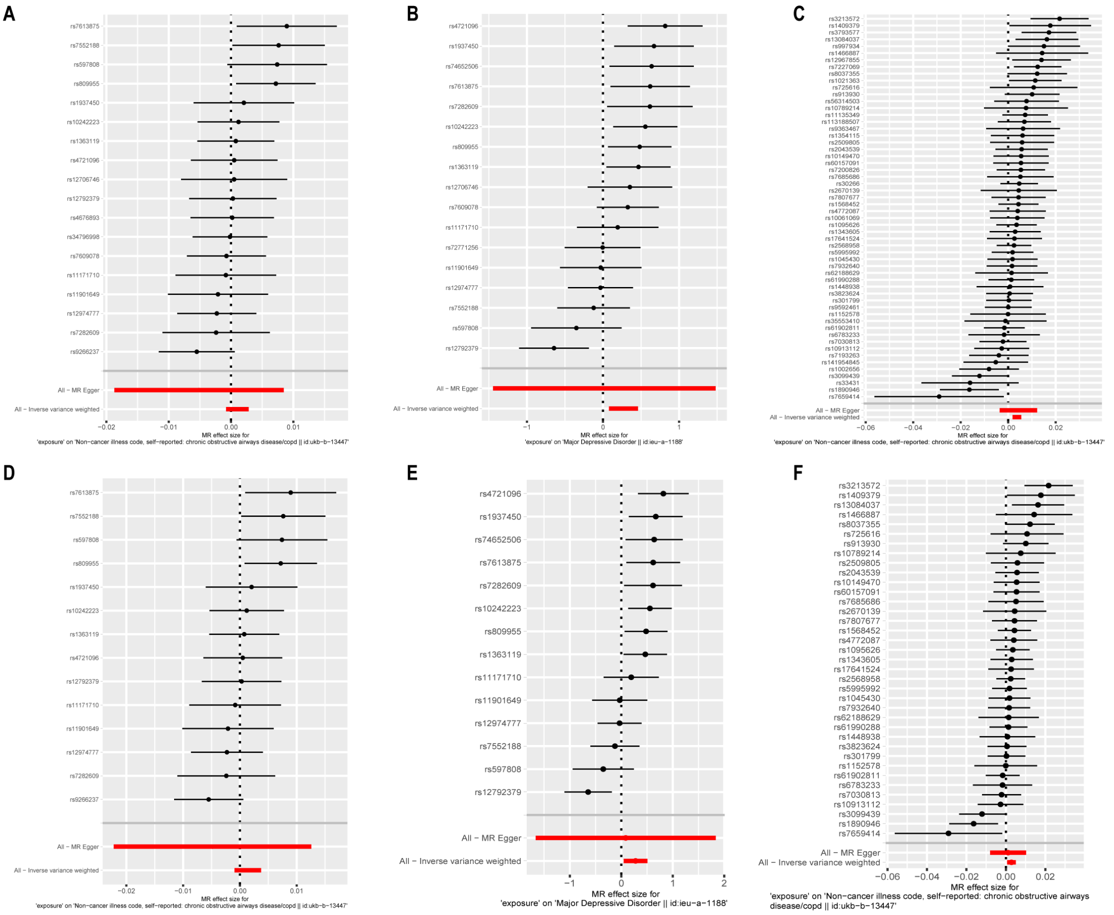

Supplementary Figure 2. Forest plot for the forward MR analysis. **(A)** GERD-COPD in MR analysis. **(B)** GERD-MDD in MR analysis. **(C)** MDD-COPD in MR analysis. **(D)** GERD-COPD in eQTL-MR analysis. **(E)** GERD-MDD in eQTL-MR analysis. **(F)** MDD-COPD in eQTL-MR analysis.

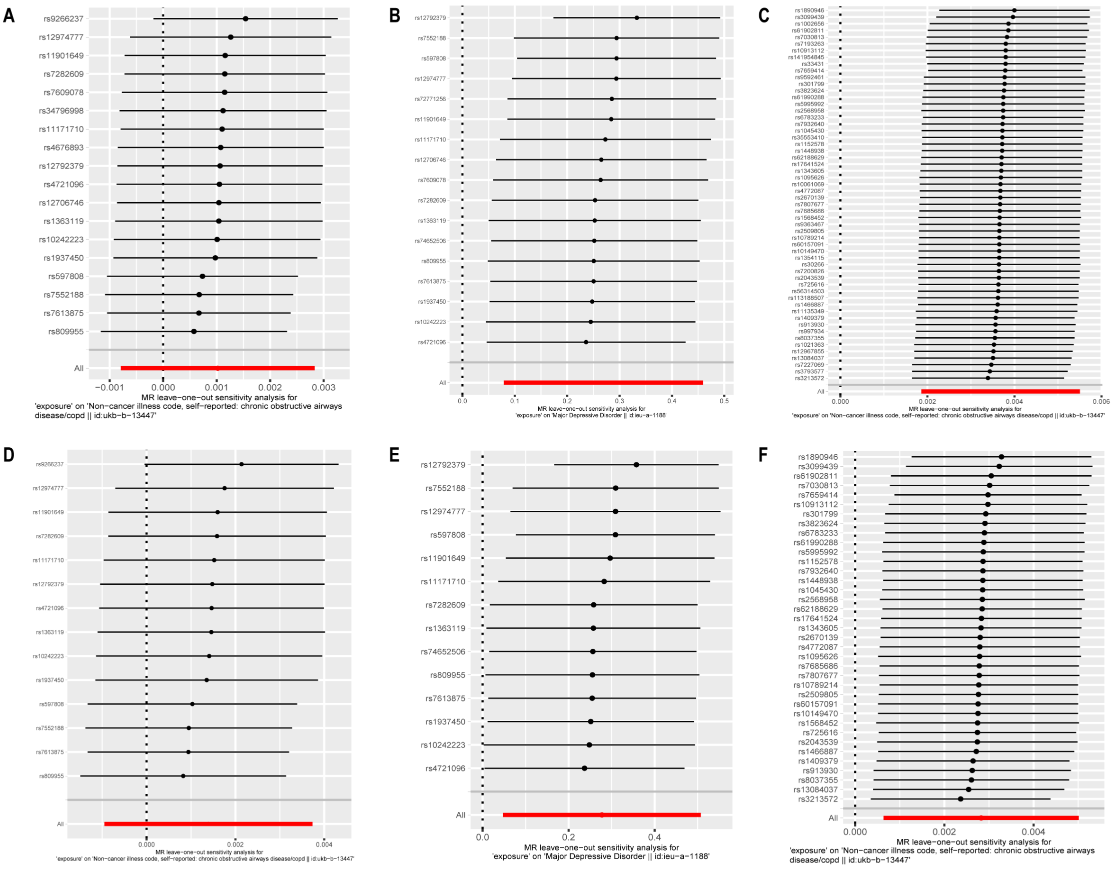

Supplementary Figure 3. Leave-one-out analysis for the forward MR analysis. **(A)** GERD-COPD in MR analysis. **(B)** GERD-MDD in MR analysis. **(C)** MDD-COPD in MR analysis. **(D)** GERD-COPD in eQTL-MR analysis. **(E)** GERD-MDD in eQTL-MR analysis. **(F)** MDD-COPD in eQTL-MR analysis.

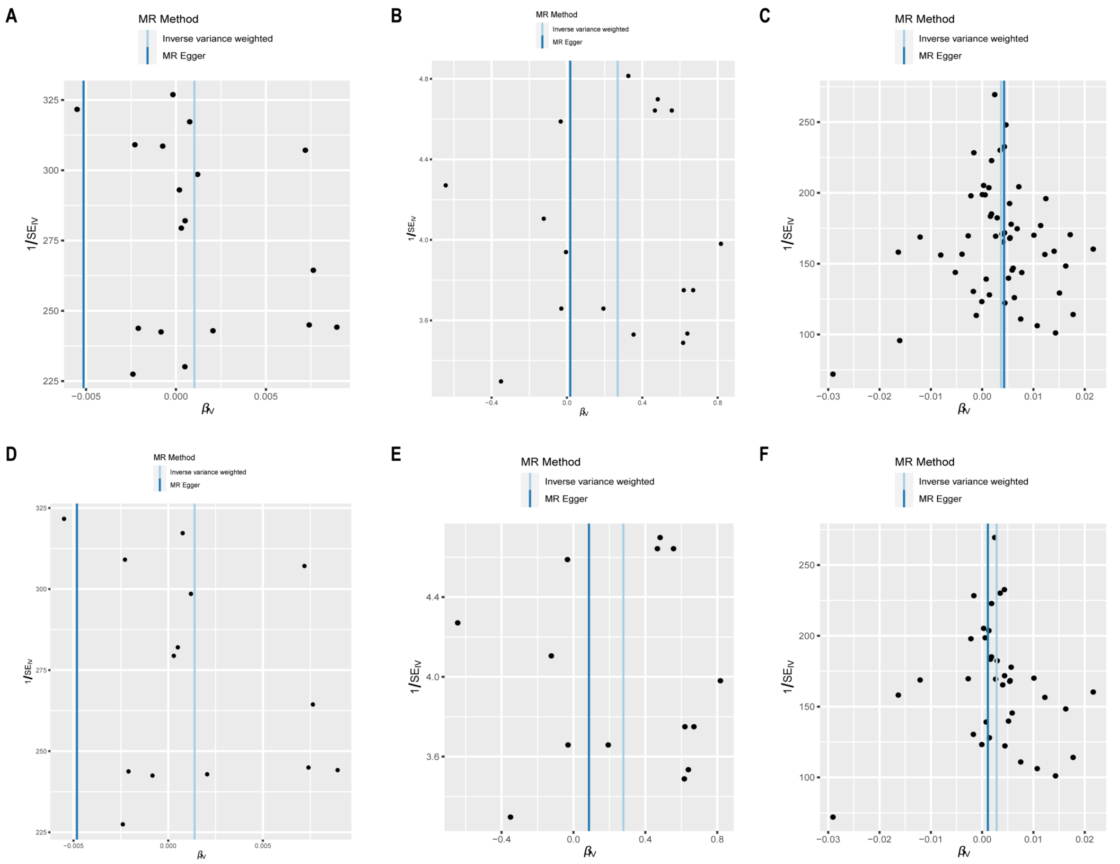

Supplementary Figure 4. Funnel plot for the forward MR analysis. **(A)** GERD-COPD in MR analysis. **(B)** GERD-MDD in MR analysis. **(C)** MDD-COPD in MR analysis. **(D)** GERD-COPD in eQTL-MR analysis. **(E)** GERD-MDD in eQTL-MR analysis. **(F)** MDD-COPD in eQTL-MR analysis.

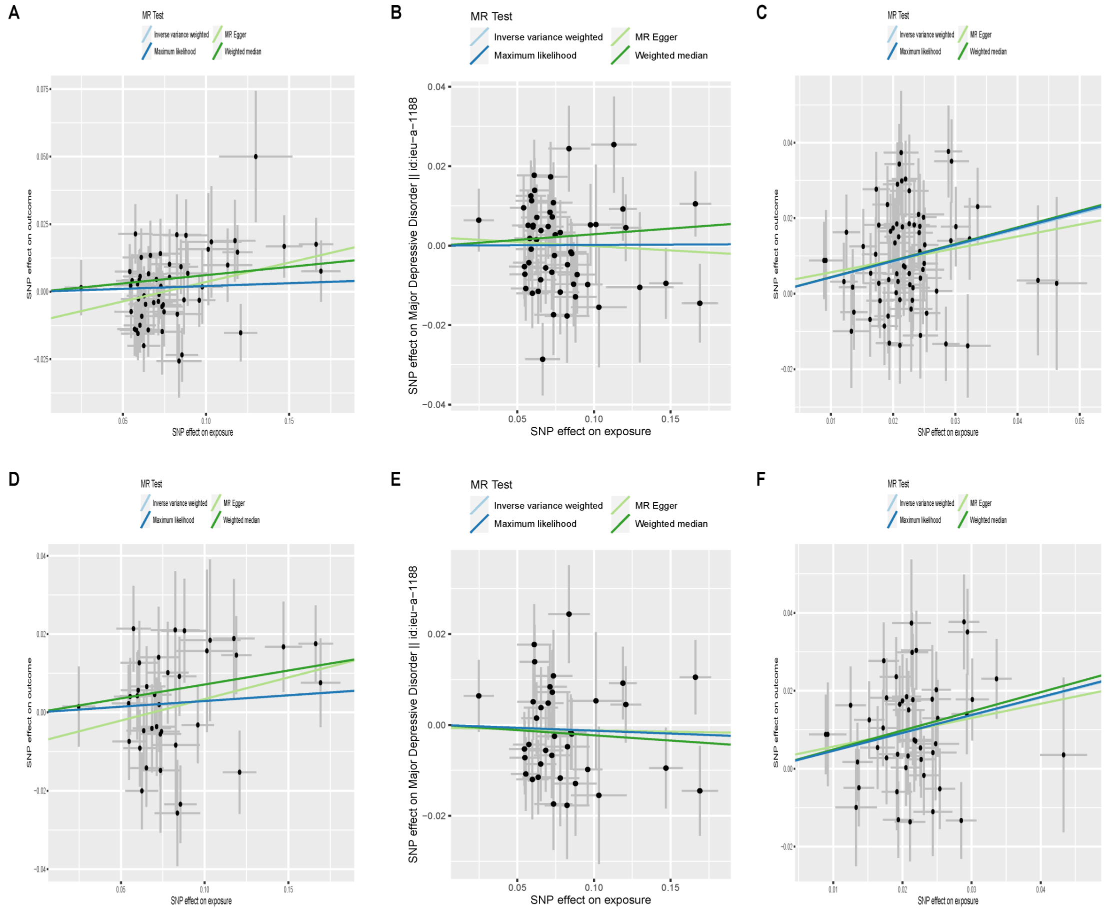

Supplementary Figure 5. Scatter plot for the reverse MR analysis. **(A)** COPD-GERD in MR analysis. **(B)** COPD-MDD in MR analysis. **(C)** MDD-GERD in MR analysis. **(D)** COPD-GERD in eQTL-MR analysis. **(E)** COPD-MDD in eQTL-MR analysis. **(F)** MDD-GERD in eQTL-MR analysis.

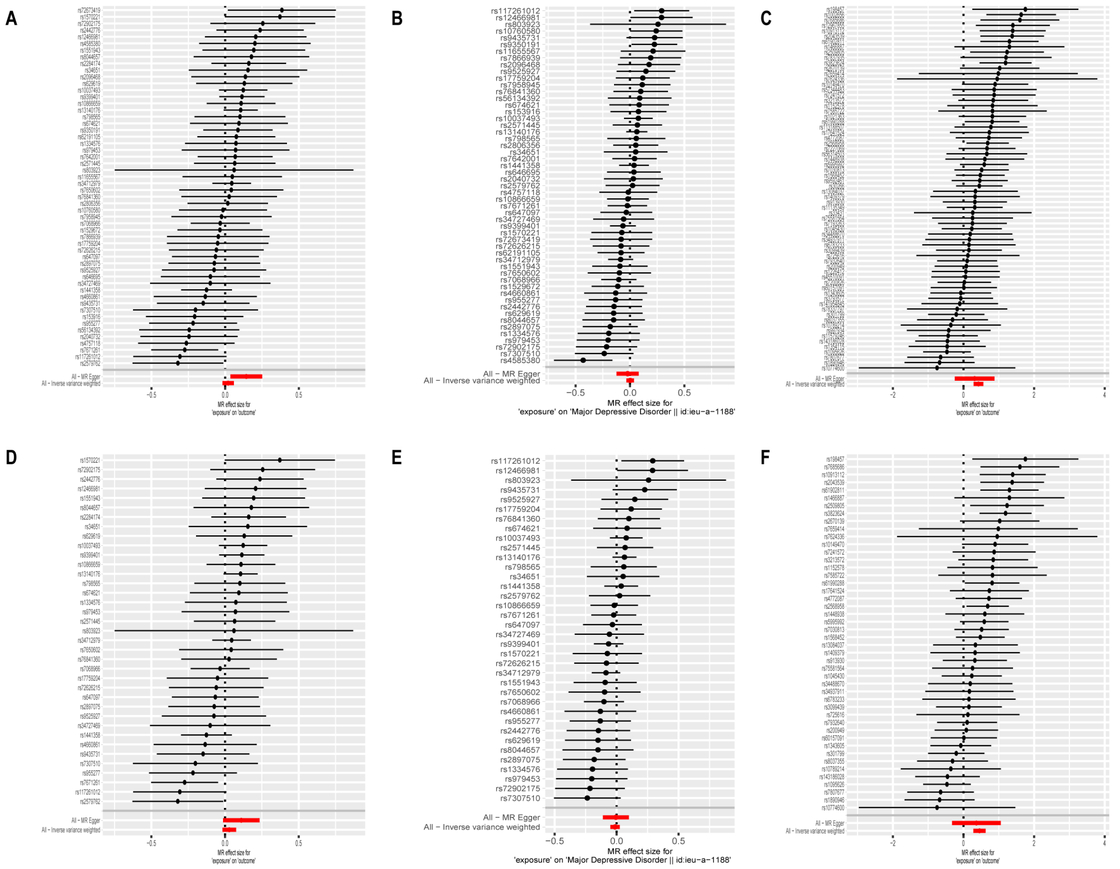

Supplementary Figure 6. Forest plot for the reverse MR analysis. **(A)** COPD-GERD in MR analysis. **(B)** COPD-MDD in MR analysis. **(C)** MDD-GERD in MR analysis. **(D)** COPD-GERD in eQTL-MR analysis. **(E)** COPD-MDD in eQTL-MR analysis. **(F)** MDD-GERD in eQTL-MR analysis.

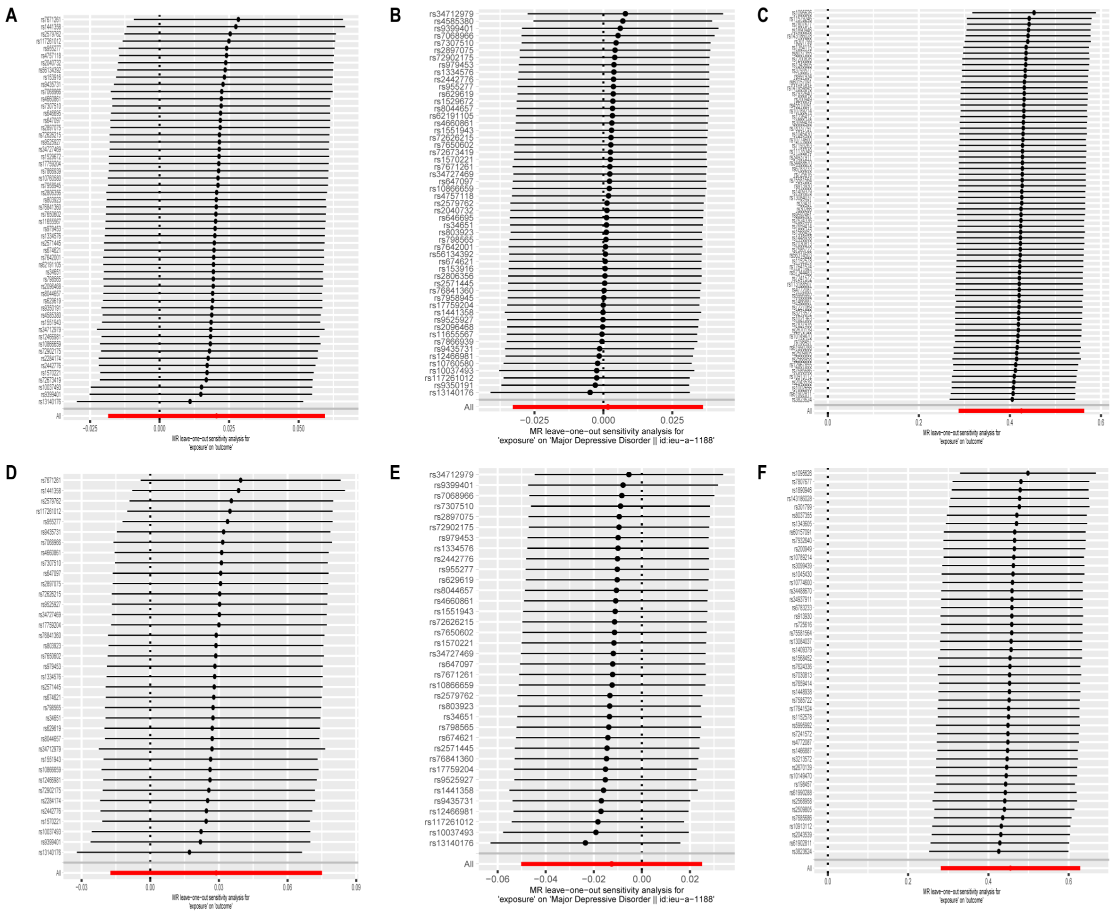

Supplementary Figure 7. Leave-one-out analysis for the reverse MR analysis. **(A)** COPD-GERD in MR analysis. **(B)** COPD-MDD in MR analysis. **(C)** MDD-GERD in MR analysis. **(D)** COPD-GERD in eQTL-MR analysis. **(E)** COPD-MDD in eQTL-MR analysis. **(F)** MDD-GERD in eQTL-MR analysis.

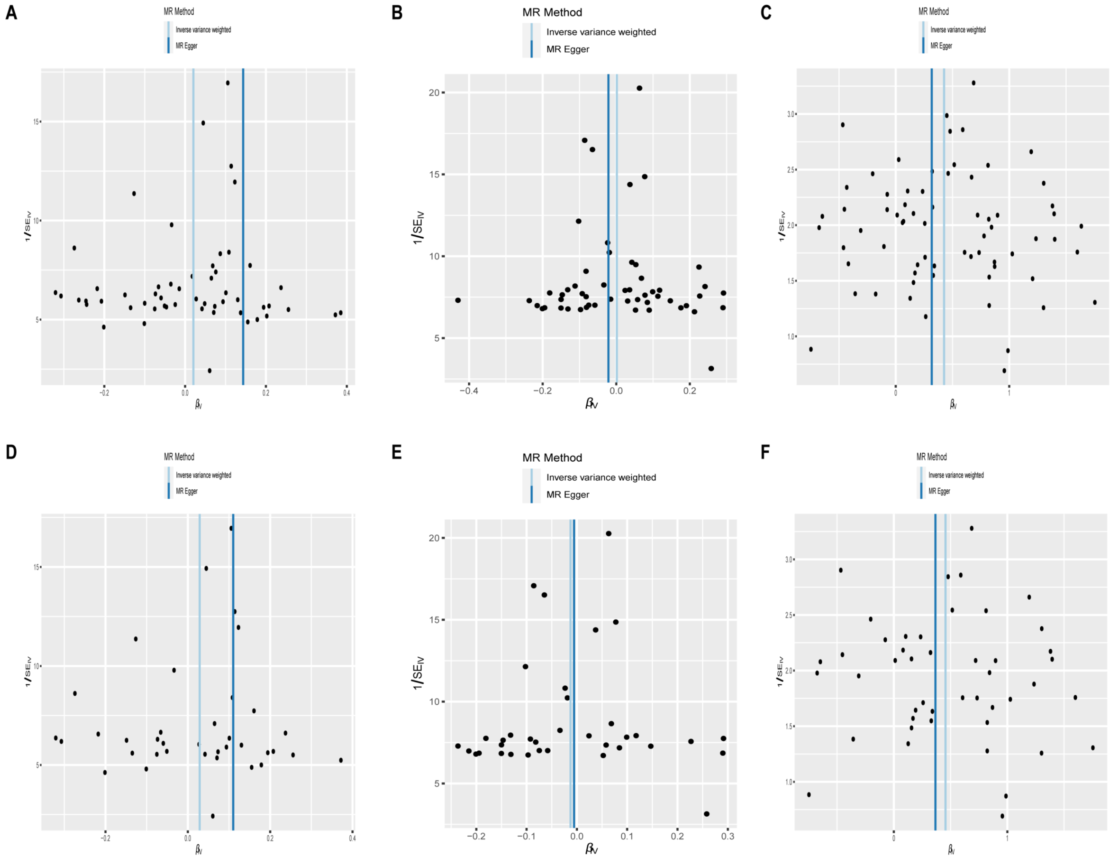

Supplementary Figure 8. Funnel plot for the reverse MR analysis. **(A)** COPD-GERD in MR analysis. **(B)** COPD-MDD in MR analysis. **(C)** MDD-GERD in MR analysis. **(D)** COPD-GERD in eQTL-MR analysis. **(E)** COPD-MDD in eQTL-MR analysis. **(F)** MDD-GERD in eQTL-MR analysis.
